# Protocol for Increasing the Sensitivity of MS-Based Protein Detection in Human Chorionic Villi

**DOI:** 10.3390/cimb44050140

**Published:** 2022-05-09

**Authors:** Timur Shkrigunov, Pavel Pogodin, Victor Zgoda, Olesya Larina, Yulia Kisrieva, Maria Klimenko, Oleg Latyshkevich, Peter Klimenko, Andrey Lisitsa, Natalia Petushkova

**Affiliations:** 1 Center of Scientific and Practical Education, Institute of Biomedical Chemistry, 119121 Moscow, Russia; lisitsa060@gmail.com; 2 Laboratory of Structure-Function Based Drug Design, Institute of Biomedical Chemistry, 119121 Moscow, Russia; pogodinpv@gmail.com; 3 Laboratory of Systems Biology, Institute of Biomedical Chemistry, 119121 Moscow, Russia; victor.zgoda@gmail.com; 4 Laboratory of Microsomal Oxidation, Institute of Biomedical Chemistry, 119121 Moscow, Russia; larina.ibmc@gmail.com (O.L.); juliaks@bk.ru (Y.K.); cyp450@mail.ru (N.P.); 5 Center for Family Planning and Reproduction, Moscow Department of Health, 117209 Moscow, Russia; klimma@yandex.ru (M.K.); latishkevich2003@mail.ru (O.L.); 6 Department of Obstetrics and Gynecology, Pirogov Russian National Research Medical University, 117997 Moscow, Russia; pa.klimenko@mail.ru

**Keywords:** chorionic villi, SDS extracts, 1DE-gel concentration, LC–MS/MS, low-abundance proteins, elective abortion, missed abortion, PSG7, bioinformatics

## Abstract

An important step in the proteomic analysis of missing proteins is the use of a wide range of tissues, optimal extraction, and the processing of protein material in order to ensure the highest sensitivity in downstream protein detection. This work describes a purification protocol for identifying low-abundance proteins in human chorionic villi using the proposed “1DE-gel concentration” method. This involves the removal of SDS in a short electrophoresis run in a stacking gel without protein separation. Following the in-gel digestion of the obtained holistic single protein band, we used the peptide mixture for further LC–MS/MS analysis. Statistically significant results were derived from six datasets, containing three treatments, each from two tissue sources (elective or missed abortions). The 1DE-gel concentration increased the coverage of the chorionic villus proteome. Our approach allowed the identification of 15 low-abundance proteins, of which some had not been previously detected via the mass spectrometry of trophoblasts. In the post hoc data analysis, we found a dubious or uncertain protein (PSG7) encoded on human chromosome 19 according to neXtProt. A proteomic sample preparation workflow with the 1DE-gel concentration can be used as a prospective tool for uncovering the low-abundance part of the human proteome.

## 1. Introduction

In recent years, great successes have been achieved in the mass spectrometric identification of differentially regulated proteins and in discovering new proteins, biomarkers, protein modifications, and polymorphisms in various human tissues [1]. Nevertheless, problems remain regarding the analysis and characterization of low-abundance proteins, including validating so-called “missing proteins”. A missing protein is an unconfirmed genetic sequence for which a protein has not yet been detected [2]. According to the international HUPO Human Proteome Project, there are currently 1343 missing proteins with no function annotated, either predicted by bioinformatics analysis or experimentally studied. The difficulty of detecting missing proteins may be related not only to their low abundance in many tissues but also to their expression in only a few cell types in the human body. Therefore, to capture the missing proteins, the experts’ collective approach has been to study a wide range of tissues, following a targeted proteomics workflow [3]. The identification of missing proteins requires significant consideration during the targeted proteomics workflow, particularly in the sample preparation, extraction, digestion, and data analysis, to increase the incidence of identification [4].

The placenta can serve as an additional origin among intact human tissues with the ultimate aim of uncovering the low-abundance part of the human proteome. The fetal part of the placenta is known as the chorion and is formed by the trophoblast. The key function of the placenta is to connect the developing fetus to the mother. One of the main components of the highly vascularized placenta is the trophoblast, the surface of which is covered with microvilli. The formation of chorionic villi begins at the end of the fourth week of gestation. At ten weeks, the microvilli are long (measuring approximately 1.5 µm), becoming shorter as pregnancy progresses, and transforming into the placenta by the sixteenth week of pregnancy. Chorionic villi are used in a prenatal test (chorionic villus sampling) that involves taking a sample of tissue from the placenta to detect specific genetic or biochemical abnormalities in an unborn baby [5]. Chorionic tissue can be obtained during a first-trimester abortion (up to the thirteenth week of pregnancy), which is a highly safe and common medical procedure [6]. According to transcriptome analysis, 65% (n = 13,074) of all human proteins (n = 20,090) are expressed in the placenta, and 288 of these genes show elevated expression in the placenta compared to other tissue types [7]. The placenta has the most enriched gene expression in common with the testis, which is a promising source of missing proteins [7,8,9].

The next step after choosing a suitable tissue for identifying the low-abundance proteins is the sample preparation procedure, an essential tool for discovering and identifying the low-abundance and missing proteins. An especially important step is protein extraction from solid tissue for subsequent mass spectrometric analysis. However, the efficient extraction of proteins of interest from tissues is not always simple. The selection of the method for cell destruction depends on the type of tissue, the amount, and the physical properties of the extracted proteins. Historically, physical lysis (mechanical disruption, sonication, and freeze/thaw cycles) was the method of choice for cell disruption and the extraction of cellular content. The main “underwater stone” is that such a strategy often involves protocols that can be difficult to reproduce due to the variability of the instruments used in different laboratories. In recent years, detergent-based techniques have become a standard step in proteomic sample preparation since they have both lysing and solubilizing effects. Although detergent-based cell lysis is an alternative to the physical disruption of cell membranes, it is often used in combination with some physical methods (homogenization and sonication) [10]. Furthermore, due to the vast number of detergents available today, choosing an appropriate one for proteomic studies is challenging. Finally, there is no ideal detergent for all applications, and the results often vary for the same application in the case of different tissues [11].

For the efficient solubilization of tissue proteins, high concentrations (0.5–4%) of detergents (for example, sodium dodecyl sulphate (SDS)) [12,13] or chaotropic agents (such as urea) [14,15] are typically required; however, these may inhibit trypsin activity, suppress LC–ESI–MS ionization, and generate high-abundance ions that interfere with mass spectrometric analysis [16]. Thus, since the composition of the extraction buffer may have a significant effect on the results, its elimination is important for subsequent proteome profiling. Therefore, the detergents’ removal must be included in the proteomic sample preparation workflow. Methods such as acetone precipitation, desalting spin columns, ultrafiltration, and molecular-weight cut-off filters are currently employed to eliminate the detergents from protein or tryptic solutions [17,18,19,20]. Nevertheless, such techniques have some known limitations, including their significant time consumption, laboriousness, and the use of organic solvents before a concentrated extract suitable for analysis can be obtained [21].

The elaboration of faster analytical sample preparation methods can be a valuable contribution to proteome research since even a reduction in the preparation time can increase sample throughput. This manuscript introduces a purification procedure for removing detergent from SDS-containing protein extracts prior to protease digestion. Different variants of this technique are currently employed to circumvent known shortcomings [22,23]. We used SDS-PAGE without protein separation in a resolving gel (which we called “1DE-gel concentration”) to reduce the complexity and cost of one-dimensional electrophoresis (1DE). Such a modification may be seen as the truncation of the SDS-PAGE stage with the isotachophoresis of biomacromolecules having similar charge-to-mass ratios due to the presence of SDS [24]. The modification of the truncated SDS-PAGE was shown to be applicable as a preparative method focused on sample cleanup and the preservation of protein content in proteomics [25,26] and useful in the concentration and isolation of DNA [27]. Unlike the classic SDS-PAGE, which produces many protein bands separated by molecular weight, a single band is obtained after the 1DE-gel concentration (truncated/shortened SDS-PAGE). The obtained single protein band includes almost all the proteins of the examined tissue after SDS solubilization, and the number of samples for subsequent enzymatic digestion and mass spectrometric analysis may be decreased. The proposed 1DE-gel concentration simplifies the sample preparation procedure by reducing the time required and its laboriousness. Furthermore, our approach increases the throughput of proteomic studies, as well as the results’ reproducibility and reliability.

To characterize the human chorionic villus proteome using LC–MS/MS, in addition to SDS-based extraction followed by 1DE-gel concentration, urea-based [28] extracts were also analyzed. Gel-based digestion increases the number of protein identifications in the human placental proteome compared to liquid digestion [14]. Nevertheless, the urea–thiourea protein extraction method remains a well-established and frequently used approach for proteomic studies of the human placenta [14,15,29,30,31]. Therefore, our study aimed to determine whether SDS-based solubilization combined with the 1DE-gel concentration sample preparation procedure and in-gel digestion led to the reproducible and relatively complete identification and quantification of the proteins in chorionic villi obtained during a first-trimester abortion at 7 to 12 weeks of normal gestation (elective abortion), as well as those obtained from a missed abortion (a nonviable intrauterine pregnancy in which the fetus is not growing normally or dies, but the placenta and embryonic tissues are still in the uterus).

## 2. Materials and Methods

### 2.1. Reagents

Tris(hydroxymethyl)aminomethane (Tris, No. 77-86-1), NaCl (No. S7653-250G), urea (No. 124-43-6), thiourea (No. 62-56-6), protease inhibitor E64 (No. 124-43-6), bicarbonate ammonium (No. 09830), acetonitrile (No. 34998-6X1L-M), iodoacetamide (IAA, No. I6125), 4-vinylpyridine (No. V3204), trifluoroacetic acid (No. 76-05-1), bicinchoninic acid (No. D8284), and bovine serum albumin (BSA, No. H4522) were purchased from Sigma-Aldrich (St. Louis, MO, USA). Sodium dodecyl sulfate (SDS, No. V6551), dithiothreitol (DDT, No. V3151), and Pierce MS Grade Trypsin Protease (No. V5113) were from Thermo Scientific Promega (Madison, WI, USA). Disodium ethylenediaminetetraacetate dehydrate (EDTA, No. 60-00-4) was from BioChemica Applichem (Darmstadt, Germany). Phenylmethylsulfonyl fluoride (PMSF, No. 36978) was from Thermo Scientific (Waltham, MA, USA). Coomassie Brilliant Blue R-250 (No. 191490250) and glycerol (No. 184695000) were from Acros Organics (Geel, Belgium). Tris(2-carboxyethyl)phosphine hydrochloride (TCEP, No. HR2-801) was from Hampton Research (Aliso Viejo, CA, USA).

### 2.2. Clinical Specimen Collection and Preparation

Human chorionic villi samples were collected at the Family Planning and Reproduction Center of the Moscow Healthcare Department (Moscow, Russia). Patient recruitment and sample collection protocols were approved by the local ethics rules of the Pirogov Russian National Research Medical University (Moscow, Russia). Informed consent was obtained from all patients. The members of the elective abortion group had no obstetric complications or history of missed abortion and had at least one child. The diagnosis of the missed abortion was based on the transvaginal ultrasound results. Abortive material at 8–11 weeks of gestation was collected transcervically, placed immediately on ice, and, within 40–50 min after termination, was processed further. Fetal-derived chorionic villi were obtained under binocular magnifying glasses, washed three times in 0.9% NaCl, dried with paper, and stored at −80 °C until use. In total, five samples were obtained from the patients after elective abortion, and three were from the patients with missed abortion.

### 2.3. Protein Extraction

After defrosting, a total of 100 mg of each chorionic villi sample was picked out with tweezers, frozen–thawed twice in liquid nitrogen, and processed using two protocols:

Protocol 1—chorionic villi placed into 500 μL of 2% SDS (Protocol 1.1) or 4% SDS (Protocol 1.2) in 100 mM Tris-HCl (pH = 7.4), 120 M NaCl, 5 mM EDTA, and 1% PMSF. The samples were manually homogenized in a Dounce glass tissue homogenizer. After sonication (BANDELIN Sonopuls HD 2070, Berlin, Germany) in an ice-cold bath for two cycles for 50 s, with 25 s intervals to reduce overheating, samples were incubated for 30 min at +40 °C in an orbital shaker ELMI (Riga, Latvia) with a platform rotation speed of 1000× *g*. After heating at 95 °C for 4 min and centrifuging (Hettich Mikro 12–24 Zentrifuge, Tuttlingen, Germany) at 14,000× *g* for 20 min (+40 °C), lysate 1 was collected, and the procedure was repeated from the sonication step. Lysate 1 and lysate 2 were combined and centrifuged at 14,000× *g* for 60 min (+40 °C), and the finished supernatant was collected.

Protocol 2—chorionic villi placed into 180 μL of 7 M urea, 2 M thiourea, 65 mM DDT, and 1% protease inhibitor E64 (freshly prepared every time). After homogenization in a Potter glass tissue homogenizer, the samples were incubated for 30 min at +40 °C with shaking on vortex every 10 min and sonicated (BANDELIN Sonopuls HD 2070) in an ice-cold bath three times for 50 s, with 25 s intervals to reduce overheating. The urea–thiourea lysate was centrifuged at 15,000× *g* at room temperature for 15 min twice to remove debris.

The total protein concentration of chorionic villi extracts was determined by the bicinchoninic acid assay [32], using BSA as a standard, on an Agilent 8453 UV–visible spectrophotometer.

### 2.4. 1DE-Gel Concentration and In-Gel Digestion

Proteins were concentrated by 1DE in denaturation conditions [33] using Protean II xi Cell (Bio-Rad, Hercules, CA, USA) to remove SDS. SDS-containing protein lysate of chorionic villi (150 μg of total protein) was mixed with the sample buffer (100 mM DTT, 4% SDS, 10% glycerol) up to 75 μL, incubated for 5 min at 95 °C, and deposited onto polyacrylamide stacking gel (4% T) (in triplicate, 50 μg of protein per gel lane). Electrophoresis (50 V, 45 min) was terminated before the migration of Bromophenol blue in the resolving gel (12%T). A single protein band was visualized by staining with Coomassie Brilliant Blue R-250 and then completely cut out and destained. Each gel band was further diced into 1 mm^3^ cubes using a scalpel, and in-gel digestion with trypsin was performed according to the standard procedure [34]. Briefly, each band was incubated in destaining buffer (50% acetonitrile (*v*/*v*) in 100 mM ammonium bicarbonate, pH = 8.9) for 45–60 min at 50 °C; destaining was repeated twice. Next, each probe was reduced with 45 mM DTT at 56 °C for 60 min, alkylated with 100 mM IAA for 15 min at room temperature in the dark. After dehydration, each was subjected to in-gel proteolysis with trypsin. For this purpose, 6.3 ± 2.0 μL of trypsin solution (25 ng/μL modified trypsin in 50 mM bicarbonate ammonium) was added, depending upon its relative staining, and the mixture was incubated for 1 h at 37 °C. After this, an additional trypsin solution was added, and the mixtures were incubated for 18 h at 37 °C. Then, 15 μL of 0.7% trifluoroacetic acid was added to each gel piece and the samples were incubated for 2 h at room temperature. The mixture of proteolytic peptides from each gel band was used for LC–MS/MS analysis.

### 2.5. In-Solution Tryptic Digestion

The pair of urea–thiourea extracts (175 μg of protein) for each study group, namely elective or missed abortion, were in-solution digested in accordance with the following standard protocol [35], which is provided in the Supplementary Materials as “Supporting_Information_In-Solution_protocol.docx”.

Protein denaturation and disulfide bond reduction were performed with a solution containing 87 mM DTT and 6.7 mM TCEP in denaturation buffer (12 mM sodium deoxycholate, 2 M thiourea, 2.5 mM EDTA, and 75 mM Tris-HCl, pH = 8.5), and further incubated at 42 °C for 60 min. The reduction solution was added to each sample in a ratio of volume of reduction solution/total protein weight of 1/1 and mixed.

Then, alkylation solution (100 μL of denaturation buffer, 10 μL of 4-vinylpyridine, and 90 μL of *N,N*-dimethylformamide, pH < 9.0) was added to each sample in a ratio of volume of alkylation solution/volume of sample of 1/12 and mixed thoroughly. The reaction mixture was incubated at 20 °C, for 60 min, in a place inaccessible to daylight.

The digestion buffer containing 100 mM CaCl_2_ and 42 mM triethylammonium bicarbonate (TEAB, 42 µL) in H_2_O (water for UV, HPLC, ACS) was added (up to 100 μL). Trypsin in the ratio 1/100 (trypsin/total protein weight) was added to the sample and then it was incubated in the dark at 44 °C and 50 rpm for 120 min in a GFL Shaking Incubator 3032 (GFL, Burgwedel, Germany). Then, another ratio of trypsin was added, and the solution was incubated in the dark at 37 °C and 50 rpm for an additional 120 min in a GFL Shaking Incubator 3032. The enzymatic digestion was stopped by adding formic acid to a final concentration of 1% and then the sample was centrifuged at room temperature (30 min, 10,000× *g*). Peptide digest mixtures were analyzed without further processing using liquid chromatography coupled with LC–MS/MS.

### 2.6. LC–MS/MS Analysis

One microgram of peptides in a volume of 1–4 μL was loaded onto the Acclaim μ-Precolumn (0.5 mm × 3 mm, 5 μm particle size, Thermo Scientific, Rockford, IL, USA) at a flow rate of 10 μL/min for 4 min in an isocratic mode of Mobile Phase C (2% acetonitrile, 0.1% formic acid). Then, the peptides were separated with high-performance liquid chromatography (HPLC, Ultimate 3000 Nano LC System, Thermo Scientific, Rockford, IL, USA) in a 15-cm--long C18 column (Acclaim PepMap RSLC inner diameter of 75 μm, Thermo Fisher Scientific, Rockford, IL, USA). Next, the peptides were eluted with a gradient of buffer B (80% acetonitrile, 0.1% formic acid) at a flow rate of 0.3 μL/min. The total runtime was 90 (or 130) minutes: this included an initial 4 (or 12) min of column equilibration to buffer A (0.1% formic acid) and then a gradient from 5 to 35% of buffer B over 65 (or 95) min and 6 min to reach 99% of buffer B, flushing 10 min with 99% of buffer B and 5 min (or 7) re-equilibration to buffer A.

MS analysis was performed at least in triplicate with a Q Exactive HF-X mass spectrometer (Q Exactive HF-X Hybrid Quadrupole-Orbitrap mass spectrometer, Thermo Fisher Scientific, Rockford, IL, USA). The capillary temperature was 240 °C, and the voltage at the emitter was 2.1 kV. Mass spectra were acquired at a resolution of 120,000 (MS) in a range of 300−1500 *m*/*z*. Tandem mass spectra of fragments were acquired at a resolution of 15,000 (MS/MS) in the range from 100 *m*/*z* to *m*/*z* value determined by a charge state of the precursor, but no more than 2000 *m*/*z*. The maximum integration time was 50 ms and 110 ms for precursor and fragment ions, correspondently. The AGC target for precursor and fragment ions was set to 1106 and 2105, correspondently. An isolation intensity threshold of 50,000 counts was determined for precursor selection, and up to the top 20 precursors were chosen for fragmentation with high-energy collisional dissociation (HCD) at 29 NCE. Precursors with a charge state of +1 and more than +5 were rejected, and all measured precursors were dynamically excluded from triggering a subsequent MS/MS for 20 s.

### 2.7. Data Processing

Forty-four LC–MS/MS runs were carried out for chorionic villi extracts from both elective and missed abortions. The initial RAW files were converted to MGF files with the ProteoWizard MSConvert program [36]. Files were imported into the SearchGUI (v. 3.3.17) platform [37] and searched with X!Tandem and MS-GF+ search algorithms against the SwissProt human database (v. 1.4.2019, FASTA format) with the following search parameters: enzyme trypsin; the maximum number of missed cleavages 1; fixed modification piridylethylation of C (in-solution tryptic digestion) or carbamidomethylation of C (in-gel digestion); variable modification oxidation methionine. Parent and fragment ions were searched with tolerances of ±5 ppm and ±0.01 Da, respectively. The PeptideShaker integrator [38] was used to obtain an Excel spreadsheet file with the results.

A list of typical protein contaminants was obtained from the CRAPome database [39], a repository of negative control MS experiments. All the proteins presented in this list with a frequency of identification ≥ 0.67 were excluded from the processing. In addition, proteins with the following UniProt IDs were excluded: P01834 (Immunoglobulin kappa constant) and P02768 (Albumin).

Records on protein identification and quantification were processed and analyzed using R language [40]:

- Data manipulation and exploration—Tidyverse collection of R packages [41];
- UpSet methodology for the visualization of intersections between sets—ggupset [42];
- Hierarchal clustering for tanglegrams—hclust function natively R language [40];
- Tanglegram plots visualization—dendextend [43].

The coefficient of variation (CV) was calculated as the relation between standard deviation and mean normalized spectral abundance factor (NSAF) values. Fold change (FC) was calculated as the relation between the highest and lowest value of NSAF.

## 3. Results and Discussion

### 3.1. 1DE-Gel Concentration for Analyzing the Chorionic Villi Proteome

**Figure 1:**
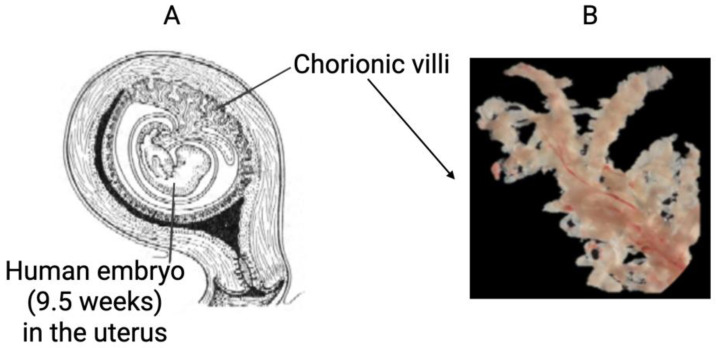
The pregnancy uterus image (**A**) and the photograph of human chorionic villi (**B**).

We employed SDS (2% or 4%) solubilization to achieve a sufficient depth of human chorionic villi proteome analysis. A clear view of the chorionic villi [44] and its location in the uterus [45] is presented in Figure 1.

**Figure 2:**
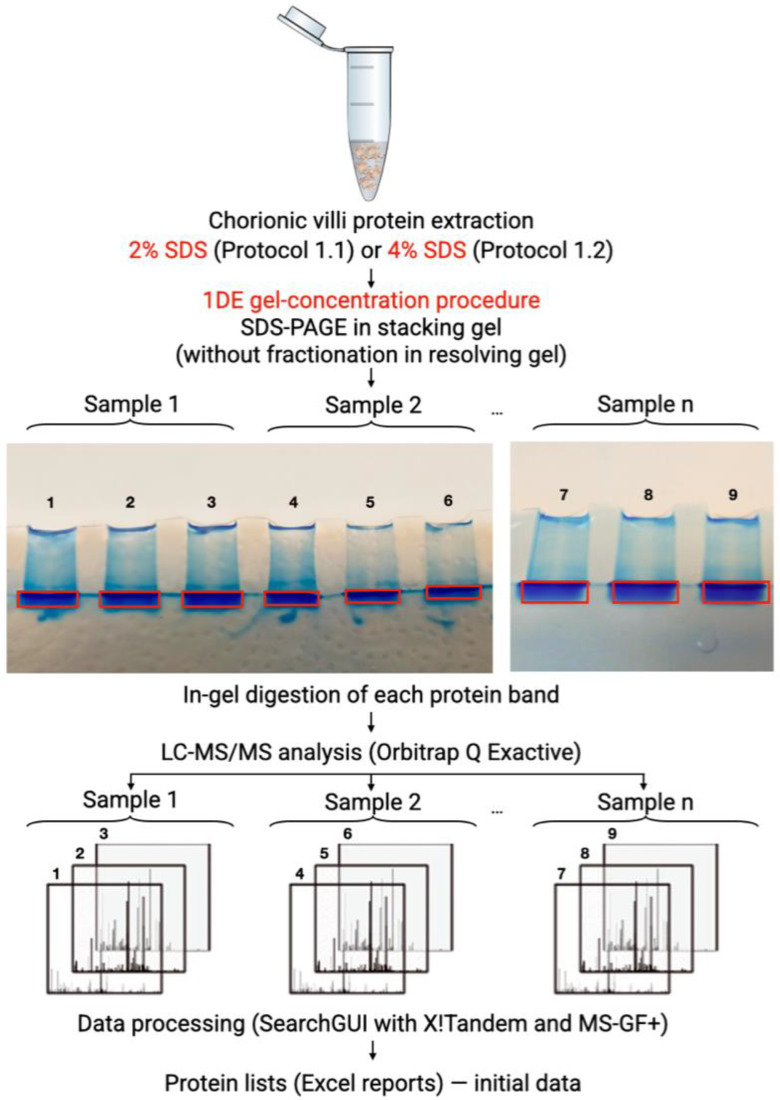
Scheme of the 1DE-gel concentration procedure. Human chorionic villi samples were solubilized in buffers based on 2% or 4% SDS and then sonicated. The obtained protein extracts were deposited onto polyacrylamide stacking gel (4%T) (in triplicate, 50 μg of protein per gel run). Electrophoresis (50 V, 45 min) was terminated before the migration of Bromophenol blue in the resolving gel. The single protein bands were excised from gel holistically and digested with trypsin. The resulting mixture of peptides was extracted for LC–MS/MS analysis.

In order to remove the SDS, we used the truncated SDS-PAGE method: the application of SDS-PAGE was limited by reaching the concentration stage in a stacking gel (4%T) without protein fractionation in a resolving gel (12%T), i.e., the 1DE-gel concentration procedure (Figure 2). The protein of interest could be absent from the identification list due to the discordance between the molecular weights (MWs) of the mass-spectrometry-detected proteins and the MWs in the resolving electrophoretic gel [46]. Each SDS-containing human chorionic villus extract was deposited onto three parallel gel runs in amounts of 50 μg per line. Electrophoresis was performed within 40–50 min at 50 V and terminated when bromophenol blue migrated into the resolving gel. As a result, we obtained one protein band on each gel line. Every protein band was entirely and holistically used for the subsequent in-gel digestion and LC–MS/MS analysis.

We performed protein extraction with urea, which is well established and frequently used in studies of the placenta proteome, for a comparative evaluation of the variability and reproducibility of the 1DE-gel concentration approach’s results. In order to obtain information about the suitability of chorionic tissue for the detection of low-abundance proteins and the possibility of using truncated SDS-PAGE to clean up the SDS (2% or 4%), chorionic villus extracts from an elective and missed abortion were analyzed. Mass spectra were recorded at different times with two to three technical replicates. The obtained LC–MS/MS data were cleaned according to the spectral matching criteria: the MGF file was excluded if its spectral criteria value was less than 5931 ± 1116. Forty MGF files based on criteria matching the spectra were selected for further data processing. These MGF files were imported into the SearchGUI platform [37], searched using the X!Tandem and MS-GF+ algorithms, and analyzed with PeptideShaker [38]. Thus, by employing chorionic villus samples after an elective and missed abortion, we identified approximately 10% of the placenta-specific proteins.

A possible limitation of the 1DE-gel concentration procedure is the potential loss of proteins with high molecular weights (MWs). The Amicon purification of SDS-based extracts can provide a protein lysate with a greater number of high-MW particles. However, the Amicon filter’s implementation may also be related to the loss of proteins [47]. Another problem with Amicon arises due to the importance of choosing the proper-size cut-off for centrifugal filters [48]. We matched the top 15 proteins with the highest molecular weights in the lists of identifications obtained by different sample preparation protocols (based on SDS or urea–thiourea). The average weights were comparable, and the maximum value was equal to 630 kDa in the case of the SDS + 1DE-gel concentration, as well as the protocol with urea that did not include electrophoresis. Thus, the 1DE-gel concentration procedure is not related to the possible removal of proteins with high molecular weights during electrophoresis.

Protein lists from the initial data (Table S1) were grouped according to their physiological origin (chorionic villi obtained after an elective or missed abortion) and sample preparation protocol (Protocols 1 and 2; Materials and Methods section). Records belonging to the distinct groups were treated as distinct datasets. The datasets and their main characteristics are shown in Table 1. We will refer to these datasets by their numbers, accordingly.

Each dataset was processed uniformly to provide a means for the strict comparison of the variability of the results obtained using different preparation procedures. The initial protein lists (Table S1) were preprocessed using the R language. Changes in the number of proteins identified and quantified relatively using the normalized spectral abundance factor (NSAF) [49], depending upon the preprocessing stage, are presented in Table 2.

As a result (Table 2), the elective abortion chorionic villi processed by 2% SDS solubilization with the 1DE-gel concentration resulted in the highest number of successful identifications (I_successful_) in comparison with 4% SDS or urea–thiourea extraction. With the missed abortion, the difference between the I_successful_ proteins in the datasets was not as expressed. The number of I_successful_ proteins was equal to 547 ± 303 or 481 ± 57 (mean ± standard deviation, n = 3) in the cases of elective or missed abortions, respectively.

Upon the completion of the proposed preprocessing (Q_reliably_), the smallest number of proteins was found in the urea–thiourea extracts (Datasets 3 and 6). The largest number of reliably quantified (Q_reliably_) proteins was observed in the samples processed with 4% SDS-based extraction buffer (Datasets 2 and 5). The presented preprocessing led to the number of Q_reliably_ proteins being equal to 131 ± 28 in each dataset (mean ± standard deviation, n = 6). Initially, the datasets had different structures (Table 1, columns “Number of samples” and “Number of replicates per sample”), which could have affected the numbers of the identified proteins (Table 2, column “P_total_”). Thus, our preprocessing strategy permitted the extraction of comparable protein lists for the further statistical analysis of overlap and variability (Table 2, column “Q_reliably_”).

Among the I_successful_ records, 32 proteins (Table S2) were involved in female pregnancy, according to the Gene Ontology database [52]. They were found in the highest numbers in the elective abortion chorion samples prepared with 2% and 4% SDS: 24 and 15 proteins, respectively. The use of the urea-containing buffer made it possible to detect only two proteins. With the missed abortion, 26 female pregnancy-involved proteins were detected; 22 and 13 of them were identified in 2% and 4% SDS extracts, respectively. In comparison, the urea-based extraction detected seven such proteins.

**Figure 3:**
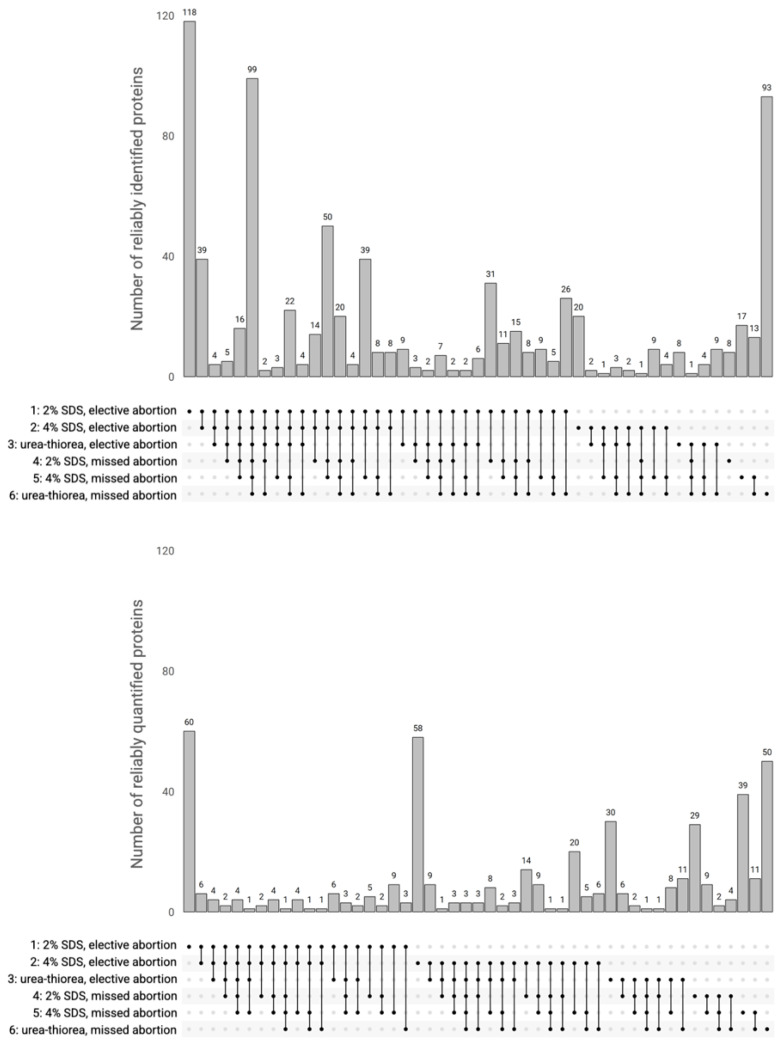
UpSet plot showing the number of shared proteins (i.e., intersections) between six human chorionic datasets. Upper panel—intersections of the reliably identified (I_reliably_) proteins; lower panel—intersections of the reliably quantified (Q_reliably_) proteins. Datasets are given as their numbers in rows of the table.

We used the UpSet plot [53] to compare six datasets based on the physiological origins of the chorionic villi and sample preparation protocols, and also to trace the connection (intersection) between the I_reliably_ and Q_reliably_ protein lists (Figure 3). Euler and Venn diagrams are among the oldest and most popular dataset visualizations [54], but they are applicable for no more than four datasets. Increases in the number of datasets make it challenging to generate intuitive diagrams that display associations among datasets, and these are not always suitable for “omics” data [55]. The UpSet plot is a widely used tool for visualizing up to 30 sets; it consists of two parts: a histogram and a table. Datasets are provided in the rows of the table. Filled-in cells in the table show which sets are the parts of an intersection, and the related histogram bars display the numbers of mutual proteins. Unconnected, single filled-in cells and the corresponding histogram bars provide information about unique proteins in the dataset. The standout high bars in Figure 3 show that only a minor overlap was apparent among the datasets. A significant number of proteins were identified and quantified in only one dataset, i.e., in one of all the possible combinations of the preparation procedure and chorionic villus origin. For the elective abortion, most unique proteins were revealed in the datasets based on the sample preparation with SDS solubilization and the 1DE-gel concentration procedure.

### 3.2. Assessment of the Variability of Protein Identification and Quantification

We proposed the 1DE-gel concentration procedure (truncated SDS-PAGE) for in-depth human chorionic villus proteome analysis. Since each biological sample was applied simultaneously on three parallel gel runs, we conducted a variability assessment for protein identification and quantification. For the variability assessment, the protein lists were determined in the “Dataset_Sample_Replicate” format. For example, the protein list of Dataset 1, Sample 1, Replicate 1 (2% SDS, elective abortion) was codified as “1_1_1”; Dataset 2, Sample 1, Replicate 2 (4% SDS, elective abortion) was codified as “2_1_2”, and so on.

**Figure 4:**
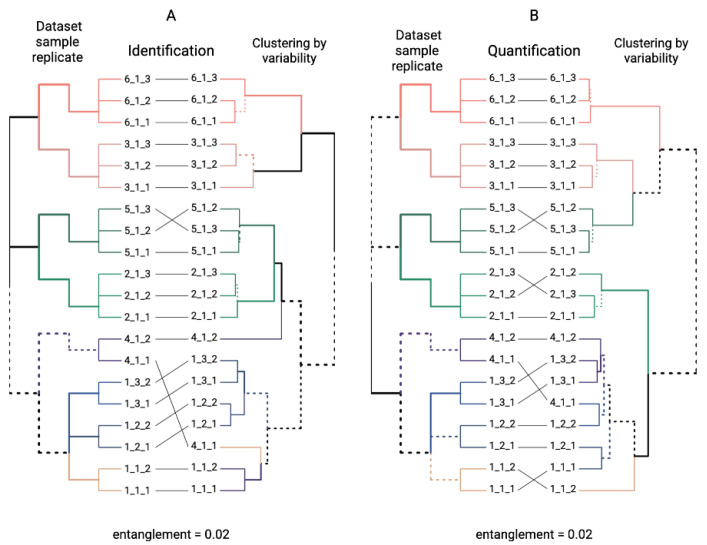
Tanglegrams comparing manually set initial “Dataset_Sample_Replicate” distribution and hierarchical clustering tree of variability measurements. Hierarchical clustering was performed by variability values for successful identification (**A**) and reliable quantification (**B**) protein lists. Individual clusters are set in different colors. Branches, which differ between two trees, are given as dashed lines.

We built hierarchical clustering trees [56] using the variability between pairs of the I_successful_ and Q_reliably_ protein lists in the “Dataset_Sample_Replicate” format. Tanglegram plots [57] were employed to compare the mentioned variability clustering (the right part of the tanglegram, Figure 4A,B) and manually set the initial “Dataset_Sample_Replicate” distribution (the left part of the tanglegram, Figure 4A,B). The entanglement values characterize the alignment quality for two given trees (right and left parts of the tanglegrams, Figure 4 A,B) and vary from 0 to 1; the lower the entanglement value, the better the alignment. As shown in Figure 4A (for I_successful_ proteins) and Figure 4B (for Q_reliably_ proteins), in both cases, the entanglements equal 0.02. This shows the severe dependencies between the LC–MS/MS analysis results and the classification with the sample preparation protocol, sample number, and replicate number. In addition, the differences between the identification and quantification protein lists are low. Given the number of existing replicates and samples, the sample preparation protocol may influence the results more than the different physiological origins of the chorionic villi. In other words, the sample preparation method (Protocol 1 and Protocol 2) was more statistically important than the sample’s origin in our processing conditions. Therefore, missed and elective samples can be considered biological replicates; this property was used in further statistical analyses. For complex types of biomaterial, researchers sometimes face the problem of insufficient numbers of biospecimens for proteomic analysis. In the present study, a shortcoming was that, for some samples, only a single probe was reported, while, for other datasets, two or three specimens were used. Such a shortcoming of our experimental design was related to the severe difficulties in the collection, long-term storage, and preparation of chorionic villus specimens for mass spectrometry. To reduce the impact of technical difficulties and to smooth out the unequal number of specimens, we used a rigorous procedure for the data preprocessing (Table 2).

The next step was to assess the significance of the differences between the protein identification/quantification variability achieved with sample preparation Protocol 1 and Protocol 2. For the comparative statistical analysis of variability, we chose Datasets 2 and 5 (4% SDS-containing buffer) and 3 and 6 (urea–thiourea-containing buffer). These datasets had an equal number of biological samples and replicates, represented chorionic villi at elective and missed abortions (biological repeats), and were processed with sample preparation Protocol 1 and Protocol 2. Since there were no indications for the data to be distributed normally and because our samples contained fewer than 30 observations, a nonparametric Wilcoxon rank-sum test [58] was used. The significance level was defined as *p* = 0.05. The Wilcoxon test statistics (W) were equal to 13 (*p* = 0.485) and 7 (*p* = 0.090) in the cases of identification and quantification variability, respectively (critical value W = 5). As the calculated *p* values exceeded the defined significance level, there was no statistically significant difference in the variability between Protocol 1 and Protocol 2. Therefore, under the conditions used, the reproducibility of protein identification and quantification achieved with sample preparation Protocol 1 corresponded to those of the well-established and frequently used urea-based approach (Protocol 2). Despite simplifying the classic SDS-PAGE protocol, the 1DE-gel concentration procedure preserves enough of the protein identification/quantification variability characteristics.

### 3.3. The 1DE-Gel Concentration for Detecting Low-Abundance Proteins in the Placenta

The examination of unusual organs, tissues, or cell types may be a prospective approach in human proteome analysis for detecting low-abundance proteins and discovering missing proteins [2,59]. In accordance with transcriptome analyses, 3.5% of the human proteome is expressed at such low concentrations in the placenta/trophoblast [7] that the analytical equipment—in many cases, mass spectrometers—cannot detect it. Thus, we believe that chorionic villi can serve as one type of human tissue for the detection and identification of such proteins.

Reliably quantified low-abundance proteins were compared with the placental trophoblastic cell protein lists that demonstrated low expression according to the Human Protein Atlas open-access database [7,60]. For this purpose, the NSAF values for Q_reliably_ proteins (Table 2) were centered and then arranged in ascending order for each dataset. Centering moves the null value, so that NSAF_1_ + … + NSAF_n_ = 0. An ascending series of centered NSAF values were then divided into quartiles to assess the proteins’ occurrence categories. Proteins with NSAF values from the first quartile (NSAF ≤ −0.021 ± 0.003, mean threshold ± standard deviation, n = 6) were taken as low-abundance proteins (LOW). Proteins with NSAF values from the second and third quartiles were assigned to the medium-occurrence protein category (MEDIUM). Proteins with NSAF values from the fourth quartile (NSAF ≥ −0.001 ± 0.007, mean threshold ± standard deviation, n = 6) were taken as high-occurrence proteins (HIGH). The division of proteins into LOW, MEDIUM, and HIGH categories is presented in Table S3. As a result, 15 proteins from the LOW category (Table 3) matched the low-expression trophoblastic proteins according to the Human Protein Atlas database [7]. Among them, ten were membrane proteins that typically occur in low abundance.

According to the Human Protein Atlas [7], our analyses mapped the low-abundance proteins, including those not previously detected by the mass spectrometry of trophoblasts. For example, using the 2% SDS-containing buffer for chorionic protein extraction with an elective abortion (dataset 1) revealed the tissue alpha-L-fucosidase protein (FUCA1). The FUCA1 protein was identified by two protein-specific peptides (Figure S1); this is typically enough to confirm the presence of a protein within a sample. FUCA1 may play an important role in the adhesion of cells during the attachment and detachment of fetal membranes [61]. Its activity in the placenta increases during embryogenesis, which is the highest during the gestational phase of 11–12 weeks, and then decreases considerably toward the end of the pregnancy.

We also detected downregulation of the reticulon-4 protein (RTN4) for the missed abortion, which is involved in the apoptotic process (GO: 0006915). The deficiency in RTN4 could lead to phenotypes such as “abnormal trophoblast layer morphology”, “embryonic growth arrest”, “decreased fetal size”, and “embryonic lethality” [62].

SDS-based extraction for the missed abortion samples (Datasets 4 and 5) allowed the detection of hyaluronan and the proteoglycan link protein 1 (HAPLN1). HAPLN1 was identified by two protein-specific peptides (Figure S2): natural ^200^*GGLDWCNAGWLSDGSVQYPITKPR* (24 aa) and natural + synthetic ^261^*FYYLIHPTK* (9 aa). HAPLN1 is essential for cartilage proteoglycan aggregate formation and has a broad spectrum of biological functions, including chondrocyte differentiation [63] and cardiac development [64]. A lack of HAPLN1 in homozygous mice resulted in perinatal lethality, accompanied by severe chondrodysplasia [63] and cardiac malformation [64].

### 3.4. The Partly Lost Pregnancy-Specific Glycoproteins

In the present study, human pregnancy-specific glycoproteins (PSGs) were identified in human chorionic villi. The human PSGs are a group of molecules almost exclusively expressed by the placental trophoblasts (chorionic villi) during pregnancy. PSGs comprise a subgroup of the carcinoembryonic antigen (CEA) family [65], an important tumor marker for colorectal and some other carcinomas [66]. PSG levels have been found to correlate well with placental function and fetal well-being [67]. Low PSG levels are associated with poor pregnancy outcomes [68].

Ten protein-coding and closely related human PSG genes (PSG1–PSG9 and PSG11) form a subgroup of the carcinoembryonic antigen (CEA) gene family on 19q13.2 [69]. Employing BLAST, we found that the PSG9 protein shares nearly 87% sequence homology with PSG1, PSG3, PSG4, and PSG6–PSG8, but only 63% with the other members of the PSG subgroup (PSG2, PSG5, and PSG11).

Using 2% SDS lysis with the 1DE-gel concentration approach for an in-depth analysis of the chorionic villus proteome allowed for the detection of all the members of the human PSG subgroup (Table 4). However, 4% SDS-based solubilization with the 1DE-gel concentration or urea-based extraction resulted in the detection of only four proteins in both cases: PSG3–PSG5, PSG9 or PSG2–PSG4, and PSG9, respectively. Among the representatives of the PSG family, we identified PSG2, PSG4, PSG6, and PSG9 in chorionic villi that were not previously detected by MS in extravillous trophoblasts, while PSG mRNA expression was reported [70].

Moreover, the use of 2% SDS-based extraction in combination with the 1DE-gel concentration led to the discovery of putative pregnancy-specific beta-1-glycoprotein 7 (PSG7), the existence of which remains uncertain (“protein uncertain”). The neXtProt platform curates protein existence (PE) evidence and assigns one of five confidence levels (PE1–5). PE1 means that there is evidence at the protein level; PE2–PE4 are missing proteins [2] (evidence at the transcript level, inferred from homology, predicted); and PE5 has dubious or uncertain evidence. PSG7 is part of the PE5 protein entries (n = 30, human chromosome 19) according to the neXtProt platform [71]. Utilizing SearchGUI, we managed to register eight peptides with a score of 100 that matched PSG7; among them, one peptide, ^256^*DVSTFTCEPK*, was unique (Figure S3, Table S4). The merging of the identifications from multiple search engines does not necessarily increase the number of reported proteins, but it does increase the number of peptides per protein and may thus generally be recommended [72]. Therefore, we used an analogue of SearchGUI, IdentiProt, based on the open-source IdentiPy algorithm [73]. IdentiProt has previously shown good identification results in analyzing human testicular tissue [74]. The search with IdentiProt allowed us to detect not only the peptide ^256^*DVSTFTCEPK* but also the second PSG7-specific peptide, ^91^*YGPAYSGR* (Figure S4, Table S5). Thus, merging the identifications from multiple search engines increased the number of unique peptides from one to two. In such a way, we were able to identify PSG7 according to two unique peptides, which were both in the detection range of 7–30 aa [75].

Employing 2% SDS-based protein solubilization followed by the 1DE-gel concentration procedure, PSG7 was identified only in chorionic villi after elective abortion. Furthermore, Su et al. [76] and Khan and Hammarström [77] discussed that the PSG7 protein might participate in gastric cancer processes and perform important functions associated with the stimulation of cell reproduction during prenatal development. Therefore, we assume that PSG7 expression in the placenta/trophoblasts is essential for the maintenance of a healthy pregnancy.

## 4. Conclusions

The non-denaturing conditions are as critical as SDS lysis; therefore, to avoid a loss of proteins due to the dilution of samples, we used only a single concentration step of SDS-PAGE. The rationality behind our idea is that the protein of interest may be absent from the identification list due to the discordance between the molecular weights (MWs) of the mass-spectrometry-detected proteins and the MWs in SDS-PAGE.

In light of the above, we highlight that a critical aspect for the overall success of the entire shotgun proteomic experiment is the optimization of each of the individual steps (e.g., protein extraction, sample preparation, and enzyme digestion). Here, we aimed to describe a bottom-up proteomics workflow, from sample preparation to data analysis. The preparation of the protein samples for mass spectrometry at each step explicitly addresses one of the several mentioned bottlenecks. We have presented an analysis of the human chorionic villus proteome using SDS-based lysis with a subsequent clean-up of the detergents. We have introduced the “1DE-gel concentration” for SDS removal as a short electrophoresis run in the stacking gel with no protein separation.

By comparing six datasets, we revealed the impact of tissue treatment and proteomic preparation protocols on the lists of identified proteins. Our preprocessing strategy permitted the extraction of comparable protein lists for further statistical analysis of the datasets’ overlap and variability. The 1DE-gel concentration procedure preserved enough of the protein identification/quantification variability characteristics for both elective and missed abortions.

The deepest coverage of the chorionic villus proteome was achieved with the 2% SDS-based lysis buffer and the 1DE-gel concentration. We mapped low-abundance proteins onto the neXtProt data for the trophoblast cells’ proteins, categorized as “low expression” or “evidence at the transcript level”. For the 1DE-gel concentration sensitivity assessment, we focused on pregnancy-specific glycoproteins (PSGs). We were able to detect all the members of the PSGs that were almost exclusively expressed by the placental trophoblasts (chorionic villi). It is noteworthy that we were able to reveal the dubious or uncertain PSG7 protein according to two unique peptides. The existence of PSG7 had not previously been demonstrated by mass spectrometry.

The 1DE-gel concentration is a method with potential applicability for the investigation of protein abundance in a range of human tissues and cell cultures (e.g., the HaCaT keratinocyte cell line; unpublished data). In summary, our results confirm that the placenta as well as chorionic villi represent a productive source for revealing downregulated and even missing proteins that can influence pregnancy and, thus, can be relevant in predefining missed abortion and inborn baby disorders.

## Figures and Tables

**Table 1:** Characterization of studied chorionic villus datasets prepared using different sample preparation protocols.

Dataset	State	Sample Preparation Protocol	Number of Samples	Number of Replicates per Sample
1	Elective abortion	2% SDS-based solubilization combined with 1DE-gel concentration and in-gel digestion (Protocol 1.1)	3	2
2	Elective abortion	4% SDS-based solubilization combined with 1DE-gel concentration and in-gel digestion (Protocol 1.2)	1	3
3	Elective abortion	Urea-based extraction combined with in-solution digestion (Protocol 2)	1	3
4	Surgically treated missed abortion	2% SDS-based solubilization combined with 1DE-gel concentration and in-gel digestion (Protocol 1.1)	1	2
5	Surgically treated missed abortion	4% SDS-based solubilization combined with 1DE-gel concentration and in-gel digestion (Protocol 1.2)	1	3
6	Surgically treated missed abortion	Urea-based extraction combined with in-solution digestion (Protocol 2)	1	3

**Table 2:** Changes in the number of proteins identified/quantified in each dataset during data preprocessing.

Dataset	P_total_	P_placenta_	I_successful_	I_proteins_	I_reliably_	Q_reliably_
1	1377	1232	884	738	591	120
2	756	690	460	379	379	176
3	594	547	298	216	216	113
4	871	782	546	299	299	112
5	764	662	449	361	361	154
6	824	723	447	368	368	110

Note: P_total_—total number of proteins mentioned in the dataset. P_placenta_—number of proteins with known transcripts in placenta, excluding common contaminants. I_successful_—number of proteins with confident identification by ≥2 with score ≥99 peptides in at least one replicate/sample. I_proteins_—number of proteins identified by ≥2 peptides in all replicates for Dataset 1 and 4, in 2/3 or more replicates for Datasets 2, 3, 5, and 6, in at least one sample. I_reliably_—number of proteins identified by ≥2 in all replicates for Dataset 1 and 4, in 2/3 or more replicates for Dataset 2, 3, 5, and 6, in at least 2/3 of samples for Dataset 1, in all samples for Dataset 2–6. Q_reliably_—number of quantified proteins with CV_NSAF_ ≤ 0.16 [50] or FC_NSAF_ ≤ 1.25 [51].

**Table 3:** The list of low-abundance proteins * that are reliably quantified in chorionic villi depends upon the tissue preparation procedure for MS/MS analysis.

##	Accession	Protein Name	Gene	Trophoblastic Cells RNA Expression HPA **	Cellular Component	Dataset
1	P01023	Alpha-2-macroglobulin	*A2M*	744.1 pTPM	secreted	1, 6
2	Q1KMD3	Heterogeneous nuclear ribonucleo protein U like 2	*HNRNPUL2*	44.6 pTPM	nucleus	1
3	P07900	Heat shock protein HSP 90-alpha	*HSP90AA1*	682.3 pTPM	cell membrane, cytoplasm, nucleus	1, 2, 4, and 5
4	Q9Y4L1	Hypoxia up-regulated 1	*HYOU1*	35.7 pTPM	endoplasmic reticulum	2, 3
5	Q03252	Lamin B2	*LMNB2*	25.5 pTPM	membrane, nucleus	2, 3
6	Q16891	MICOS complex subunit MIC60	*IMMT*	63.5 pTPM	membrane, mitochondrion	2
7	P00505	Aspartate aminotransferase, mitochondrial	*GOT2*	42.4 pTPM	membrane, mitochondrion	2
8	Q9BSJ8	Extended synaptotagmin 1	*ESYT1*	65.4 pTPM	endoplasmic reticulum	2
9	P22307	Non-specific lipid-transfer protein	*SCP2*	139.1 pTPM	cytoplasm, mitochondrion	3, 6
10	Q13813	Spectrin alpha, non-erythrocytic 1	*SPTAN1*	100.2 pTPM	cytoplasm	3
11	P02751	Fibronectin 1	*FN1*	5227.7 pTPM ***	secreted	3, 4
12	P50395	Rab GDP dissociation inhibitor beta	*GDI2*	224.3 pTPM	cytoplasm, membrane	4
13	P02452	Collagen alpha-1(I) chain	*COL1A1*	744.6 pTPM	secreted	5
14	Q8IZ83	Aldehyde dehydrogenase family 16 member A1	*ALDH16A1*	8.8 pTPM	membrane	6
15	P09110	3-ketoacyl-CoA thiolase, peroxisomal	*ACAA1*	28.6 pTPM	peroxisome	6

* according to the Human Protein Atlas organism-specific database (https://www.proteinatlas.org/humanproteome/tissue/placenta, accessed on 8 May 2022). ** The level of protein mRNA was represented by the mean protein-coding transcripts per million (pTPM). *** Medium RNA expression according to the Human Protein Atlas. The fibronectin 1 (FN1) protein is a special case: the human placenta, during the first trimester of pregnancy (9–12 weeks), expressed a medium level of FN1 (mRNA with a pTPM of 5227.7); however, FN1 demonstrated low protein expression via immunohistochemistry.

**Table 4:** The list of pregnancy-specific glycoproteins (PSGs) identified in human chorionic villi.

##	Entry	Gene Name	Protein Name	Length, aa	Mass, Da	Sample Preparation Protocol	
						Elective Abortion	Missed Abortion
1	Q00887	*PSG9*	Pregnancy-specific beta-1-glycoprotein 9	426	48,272	Protocol 1.1; Protocol 1.2; Protocol 2	Protocol 1.1; Protocol 1.2
2	Q9UQ74	*PSG8*	Pregnancy-specific beta-1-glycoprotein 8	426	47,772	Protocol 1.1	nd
3	Q13046	*PSG7*	Putative pregnancy-specific beta-1-glycoprotein 7	419	47,027	Protocol 1.1	nd
4	Q00889	*PSG6*	Pregnancy-specific beta-1-glycoprotein 6	435	48,814	Protocol 1.1	nd
5	Q15238	*PSG5*	Pregnancy-specific beta-1-glycoprotein 5	335	37,713	Protocol 1.1; Protocol 1.2	Protocol 1.1
6	Q00888	*PSG4*	Pregnancy-specific beta-1-glycoprotein 4	419	47,113	Protocol 1.1; Protocol 1.2; Protocol 2	nd
7	Q16557	*PSG3*	Pregnancy-specific beta-1-glycoprotein 3	428	47,945	Protocol 1.1; Protocol 2	Protocol 1.1; Protocol 1.2
8	P11465	*PSG2*	Pregnancy-specific beta-1-glycoprotein 2	335	37,216	Protocol 1.1; Protocol 2	Protocol 1.1
9	Q9UQ72	*PSG11*	Pregnancy-specific beta-1-glycoprotein 11	335	37,146	Protocol 1.1	nd
10	P11464	*PSG1*	Pregnancy-specific beta-1-glycoprotein 1	419	47,223	Protocol 1.1	Protocol 1.1; Protocol 1.2

Note: Protocol 1.1—2% SDS-based solubilization with 1DE-gel concentration; Protocol 1.2—4% SDS-based solubilization with 1DE-gel concentration; Protocol 2—Urea-based extraction; nd—not detectable.

## Data Availability

The data presented in this study have been deposited in the ProteomeXchange Consortium via the PRIDE partner repository, and are openly available under accession PXD024759.

## Supplementary Materials

The following supporting information can be downloaded at: https://www.mdpi.com/article/10.3390/cimb44050140/s1, Mass spectrometry data are available from the ProteomeXchange Consortium (http://www.proteomexchange.org, accessed on 8 May 2022) under accession PXD024759. Figure S1: Mass spectra and fragment ions of the peptides *DLVGELTAL* (163–173, 11 aa, natural + synthetic) and *DGLIVPIFQE* (344–353, 10 aa, natural), which are specific to the tissue alpha-L-fucosidase (FUCA1, P04066) encoded on human chromosome 1 (SearchGUI searching); Figure S2: Mass spectra and fragment ions of the peptides *GGLDWCNAGWLSDGSVQYPITKPR* (200–223, 23 aa, natural) and *FYYLIHPTK* (10 aa, natural), which are specific to the hualuronan and proteoglycan link protein 1 (HAPLN1, P10915) encoded on human chromosome 5 (SearchGUI searching); Figure S3: Mass spectrum and fragment ions of the peptide *DVSTFTCEP* (256–265, 9 aa, 592.27 m/z, charge 2+), which is specific to the putative pregnancy-specific beta-1-glycoprotein 7 (PSG7, Q13046) protein uncertain encoded on human chromosome 19, identified using SearchGUI with simultaneously integrated search algorithms X!Tandem and MS-GF+; Figure S4: Mass spectra and fragment ions of the peptides *YGPAYSGR* (91–98, 8 aa) and *DVSTFTCEPK* (255–265, 11 aa), which are specific to the putative pregnancy-specific beta-1-glycoprotein 7 (PSG7, Q13046) protein uncertain encoded on human chromosome 19 (IdentiProt searching); Table S1: Report about the proteins of human chorionic villi samples obtained after elective or missed abortion using different sample preparation protocols (Datasets 1, 4—2% SDS-based solubilization combined with the 1DE-gel concentration and in-gel digestion; Datasets 2, 5—4% SDS-based solubilization combined with the 1DE-gel concentration and in-gel digestion; Datasets 3, 6—urea-thiourea-based extraction combined with in-solution digestion); Table S2: The list of proteins which are identified by ≥2 peptides in human chorionic villi extracts, and involved in female pregnancy process according to The Gene Ontology database (https://www.ebi.ac.uk/QuickGO/GTerm?id=GO:0007565, accessed on 8 May 2022); Table S3: Division of the reliably quantified proteins into LOW, MEDIUM and HIGH occurrence categories by NSAF values; Table S4: The list of peptides which are identified by SearchGUI, and belong to putative pregnancy-specific beta-1-glycoprotein 7 (PSG7)—protein uncertain encoded on human chromosome 19; Table S5: The list of peptides which are identified by IdentiProt, and belong to putative pregnancy-specific beta-1-glycoprotein 7 (PSG7)—protein uncertain encoded on human chromosome 19; Supporting_Information_In-Solution_protocol: In-solution tryptic digestion protocol.
