# Supplementary material for: Protocol for Increasing the Sensitivity of MS-Based Protein Detection in Human Chorionic Villi
Source: Curr Issues Mol Biol. 2022 May 9;44(5):2069–88. doi: 10.3390/cimb44050140 (PMC9164042; doi:10.3390/cimb44050140)
Supplement: Supplementary file 1 [file cimb-44-00140-s001.zip › Supporting_Information_In-Solution_protocol.pdf]

Supporting Information

Protocol for Increasing the Sensitivity of MS-Based Protein Detection in Human Chorionic Villi

Timur Shkrigunov, Pavel Pogodin, Victor Zgoda, Olesya Larina, Yulia Kisrieva, Maria Klimenko, Oleg Latyshkevich, Peter Klimenko, Andrey Lisitsa and Natalia Petushkova

In-solution tryptic digestion protocol

Reagents:

- **Denaturation Buffer**: 12 mM sodium deoxycholate (DOC), 2 M thiourea, 2.5 mM EDTA, and 75 mM Tris-HCl, pH = 8.5
- **Reduction Solution** (prepare fresh before use): denaturation buffer, 87 mM dithiothreitol (DTT), and 6.7 mM tris(2-carboxyethyl)phosphine *(*TCEP)
- **Alkylation Solution** (prepare fresh before use, pH < 9.0): denaturation buffer (100 µl), 4-vinylpyridine (10 µl), and *N,N*-dimethylformamide (90 µl)
- **Digestion Buffer** (prepare fresh before use): 100 mM $CaCl_2$, 42 mM triethylammonium bicarbonate (TEAB, 42 µl) in H_2O (water for UV, HPLC, ACS)
- **Trypsin Stock Solution**: 20 ng sequencing grade modified trypsin (Promega, Madison, WI, USA) in 100 µl of manufacture buffer (can be stored at - 40 or - 80°C for several months)
- **Formic acid**

Protocol:

1. Transfer the aliquot of urea-based extract (175 µg of protein) to a tube and dry down samples in a Eppendorf Concentrator 5301 (Hamburg, Germany)
2. Add Reduction Solution
   Use reduction solution in the ratio: reduction solution/total protein weight = 1/1
3. Incubate at 42°C for 60 min

4. Add Alkylation Solution

   Use alkylation solution in the ratio: alkylation solution/V_{probe} = 1/12

5. Incubate at 20°C for 60 min in the dark

6. Add Digestion Buffer (up to 100 µl) and mix

7. Add Trypsin

   Use an enzyme in the ratio: trypsin/total protein weight = 1/100

8. Incubate at 44°C and 50 rpm for 120 min in a GFL Shaking Incubator 3032 (Burgwedel, Germany) in the dark

9. Add another portion of Trypsin

   Use an enzyme in the ratio: trypsin/total protein weight = 1/100

10. Incubate at 37°C and 50 rpm for 2 h in a GFL Shaking Incubator 3032 in the dark

11. Add formic acid to a final concentration of 1%

12. Centrifuge for 30 min at 10,000× *g*

13. Transfer the supernatant (5 µl) to Agilent clear glass vial inserts, place it into a Agilent vial and screw a cap

14. Divide the rest of the supernatant into aliquots and stored at -20°C (~ 1 month)
