# Supplementary figures and images for: Protocol for Increasing the Sensitivity of MS-Based Protein Detection in Human Chorionic Villi
Source: Curr Issues Mol Biol. 2022 May 9;44(5):2069–88. doi: 10.3390/cimb44050140 (PMC9164042; doi:10.3390/cimb44050140)

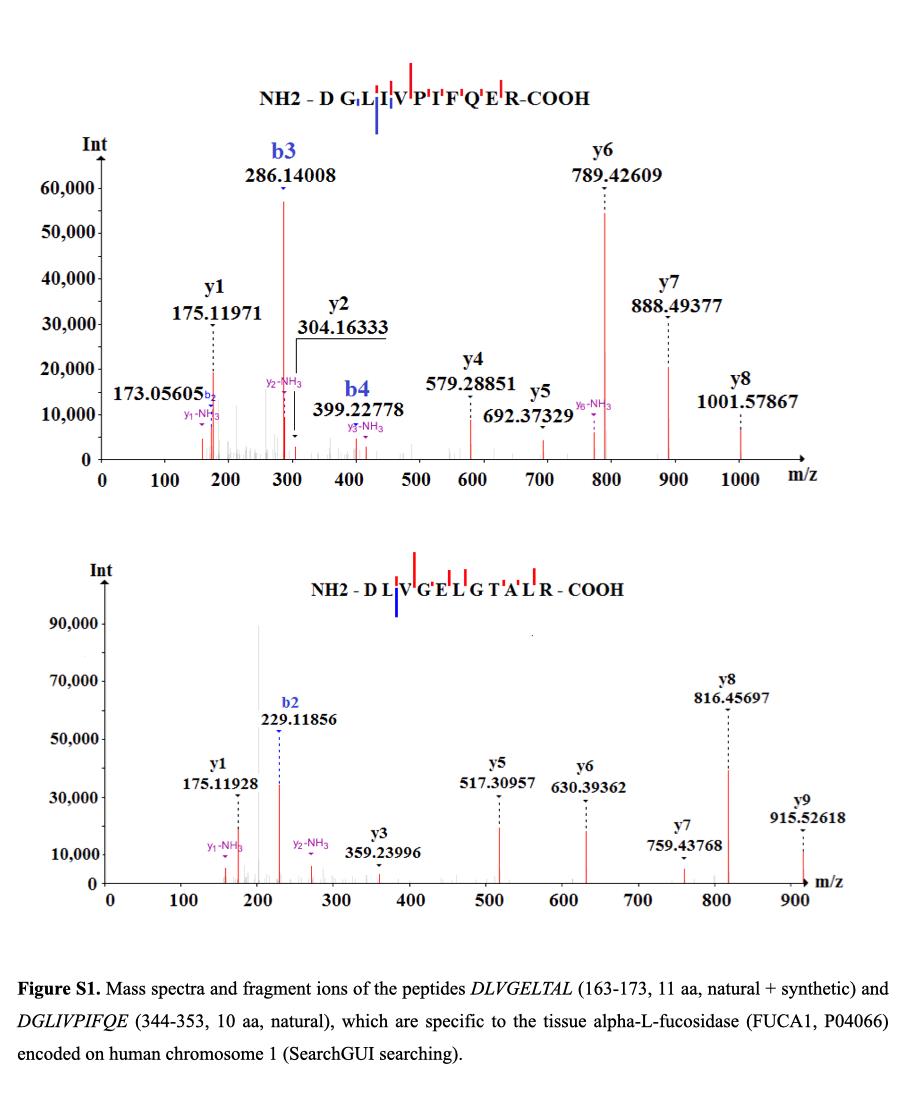

Supplement: Supplementary file 1 [file cimb-44-00140-s001.zip › Figures Supl/Figure_S1.jpg]

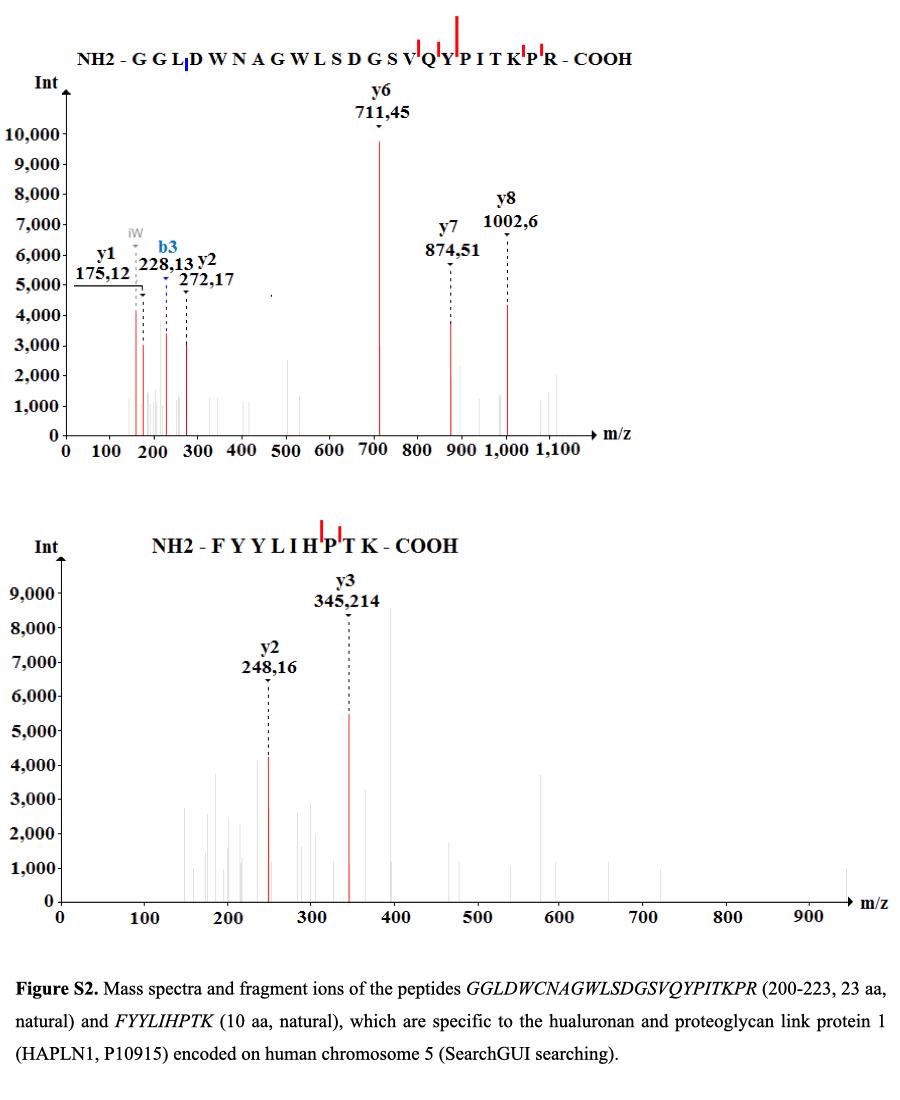

Supplement: Supplementary file 1 [file cimb-44-00140-s001.zip › Figures Supl/Figure_S2.jpg]

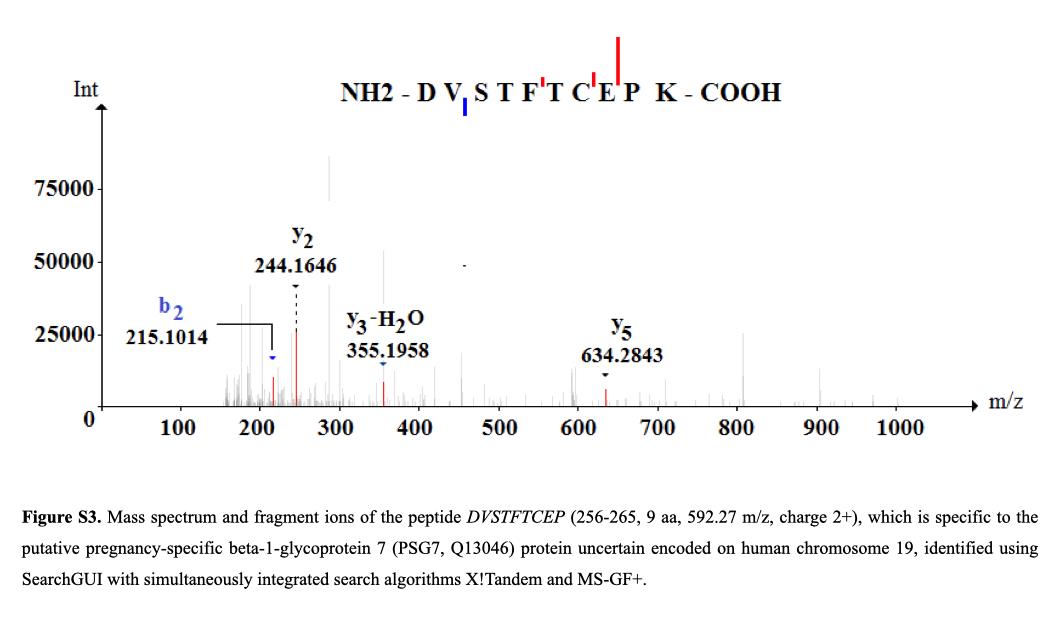

Supplement: Supplementary file 1 [file cimb-44-00140-s001.zip › Figures Supl/Figure_S3.jpg]

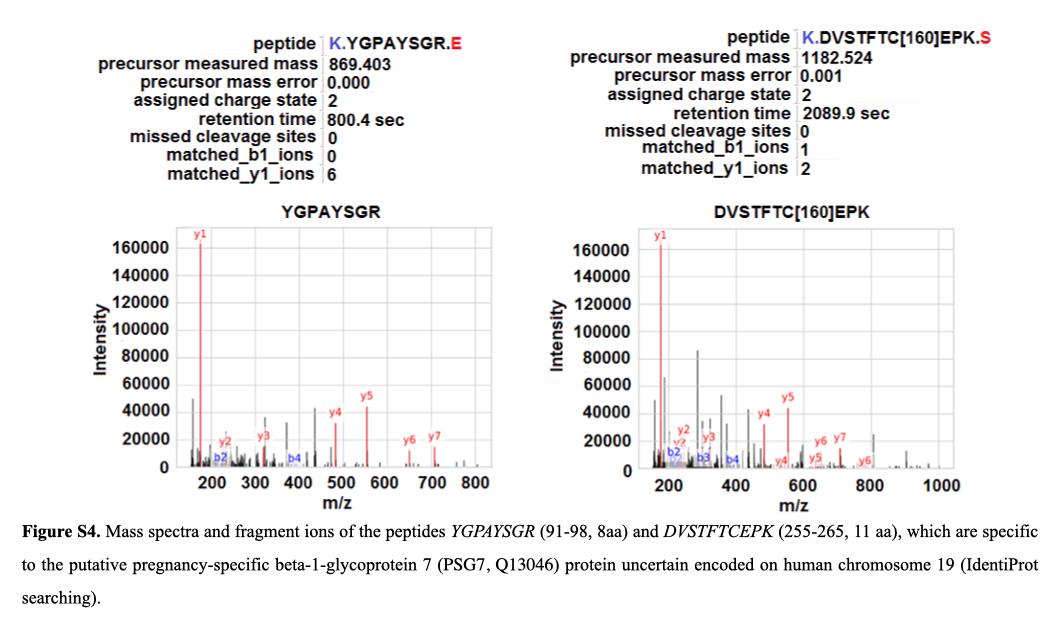

Supplement: Supplementary file 1 [file cimb-44-00140-s001.zip › Figures Supl/Figure_S4.jpg]
